# Development and validation of a magnetic resonance imaging-based model for the prediction of progression-free survival before initial treatment of hypopharyngeal cancer treated with chemoradiation

**DOI:** 10.3389/fonc.2026.1928542

**Published:** 2026-10-28

**Authors:** Hengmin Tao, Jia Wei, Qiqi Zheng, Xinbo Yang, Yahong Sun

**Affiliations:** 1 Department of Oncology, Shandong Provincial Third Hospital, Shandong University, Jinan, China; 2 Digital Resource Center, Shandong Open University, Jinan, China

**Keywords:** DWI, HPSCC, hypopharyngeal squamous cell carcinoma, progression-free survival, radiomics

## Abstract

**Objective:**

To develop a radiomics-clinical model for prediction of the progression-free survival (PFS) in hypopharyngeal squamous cell carcinoma (HPSCC) patients treated with concurrent chemoradiotherapy (CCRT).

**Materials and methods:**

One hundred and thirteen patients with hypopharyngeal cancers treated by CCRT were enrolled and randomly divided into training (N = 80) and validation (N = 33) groups. Python’s PyRadiomics toolkit was used to extract radiomic features from magnetic resonance diffusion-weighted imaging (DWI) sequences, which included four categories: shape (14), first-order (230), texture (306), and wavelet (738). All feature preprocessing steps—including normalization, intraclass correlation coefficient (ICC) filtering, Pearson correlation filtering, and feature selection—were performed exclusively within the training set, with the same preprocessing parameters subsequently applied to the validation set. The least absolute shrinkage and selection operator (LASSO) penalized Cox regression algorithm with ten-fold cross-validation was used to screen key radiomic features and construct the Rad-score. We used Kaplan-Meier analysis and log-rank tests to assess the ability of the radiomic signature to stratify PFS risk, and constructed a radiomics nomogram to predict individual PFS probabilities at 1 and 2 years.

**Results:**

The radiomics model consisting of six important features performed well in classifying patients into high- and low-risk groups of progression in both the training and test groups (log-rank test, p = 0.006 in the training cohort; p = 0.023 in the validation cohort, using the cutoff value of 0.025 determined from the training set). Age and T stage were chosen as clinically important variables based on univariate Cox regression analysis (p < 0.05). The radiomics nomogram demonstrated good differentiation ability, with C-indices of 0.904 (95% confidence interval [CI], 0.816–0.992) and 0.858 (95% CI, 0.690–1.000) in the training and validation cohorts, respectively. The time-dependent AUCs for predicting 1-year and 2-year PFS were 0.867 and 0.837, respectively, in the training cohort, and 0.737 and 0.715 in the validation cohort.

**Conclusion:**

The multiparametric DWI-based radiomics model may serve as a potential tool to predict PFS in patients with HPSCC treated with CCRT after further prospective validation.

## Introduction

Hypopharyngeal squamous cell carcinoma is a rare and approximately 80% of patients are diagnosed at an advanced stage (1, 2). Current treatment strategies are aimed at preserving laryngeal functions while maximizing long-term survival benefits (3). Chemoradiotherapy (CRT) is commonly used for advanced HPSCC because it can preserve laryngeal function better than surgery without affecting survival (4, 5). Unfortunately, the local recurrence rate is about 50% in advanced patients who receive non-surgical treatment (6), and salvage surgery may lead to complications, including wound infection, wound nonunion, pharyngeal fistula (7). Hence, developing a model for accurately predicting early progression after CRT in patients with HPSCC is clinically critical.

Diffusion-weighted imaging (DWI) on MRI has been widely used to distinguish benign from malignant tumors and evaluate tumor viability in oncology, and it can also help predict treatment response and clinical outcomes for various cancers treated with radiotherapy and CRT (8, 9). The apparent diffusion coefficient (ADC) map acquired from two DWIs (b values of 0 and 600 mm²/s, respectively) using an MRI workstation is independent of the magnetic field intensity, which can overcome the effect of T2 shine-through, allowing for more meaningful comparisons of results. ADC measurements taken before, during, and after treatment have been proven to be prognostic indicators in HPSCC (10). Radiomic signatures (RS) is a promising approach that involves the combined analysis of multiple radiomic signatures and can facilitate individualized prognostic prediction for several types of cancers (11).

Previous studies have reported correlation between HPSCC prognosis and radiomics based on CT and MRI imaging (12, 13). However, there are no studies that combine multiple image features of ADC images on MRI with RS to predict early local-regional recurrent disease in HPSCC patients after CRT.

The aim of this study was to construct a more accurate clinical-radiomic model to predict the PFS in HPSCC patients treated with CCRT, and develop individualized treatment strategies for patients in advance.

## Materials and methods

### Patient selection

We retrospectively analyzed 169 patients newly diagnosed hypopharyngeal cancer in Shandong Provincial Third Hospital from March 2016 to February 2019. The inclusion criteria included: (1) pathologically diagnosed squamous cell cancer, (2) pretreatment contrast-enhanced MRI, (3) treatment with radical CRT. A total of 113 patients were randomly allocated the training group (80 cases) and the validation group (33 cases) in a 7:3 ratio, which is presented in **Figure 1**. The training set was used exclusively for all data-dependent procedures, including feature preprocessing, normalization parameter estimation, ICC filtering, Pearson correlation filtering, LASSO-Cox feature selection, and cutoff determination. The validation set was used only once for final model evaluation and was not involved in any parameter estimation or model development steps. In accordance with national legislation and institutional requirements, this retrospective study did not require participants to sign informed consent. The waiver of informed consent was approved by the Medical Ethical Committee of Shandong Provincial Third Hospital (No. XYK20190210).

**Figure 1:**
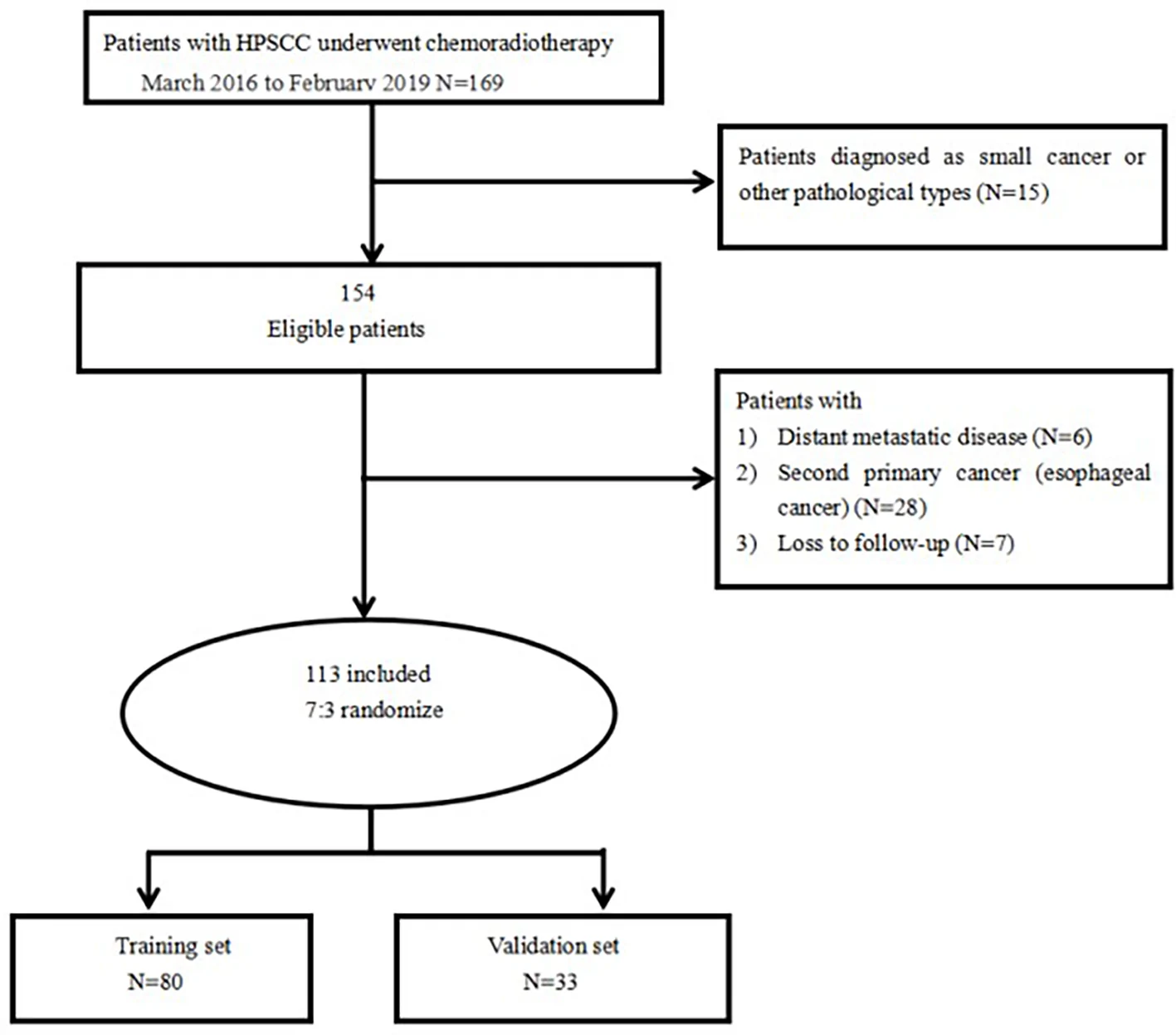
Patients recruit pathway.

### Follow-up and survival

Patients were followed up every 2 months in post-treatment year, every 3 months in year 2, and 4–6 months thereafter. Patients routinely underwent fibrolaryngoscopy and CT scans. PFS was defined as the time from the initiation of CCRT (time origin) until disease progression or death from any cause, whichever occurred first. Disease progression was defined as local recurrence, regional recurrence, or distant metastasis confirmed by imaging or biopsy. Patients who were alive without progression at the last follow-up, lost to follow-up, or who died from non-cancer causes were censored at the date of their last known follow-up. For the purpose of model evaluation, prediction horizons were set at 1 year and 2 years post-treatment.

### DWI data acquisition and tumor segmentation

All patients underwent a 3.0-T MR scanner (GE Signa HDxt) before treatment. Axial sequences were obtained from the skull base to the aortic arch. DWI was performed with a single-shot spin-echo echoplanar imaging sequence using a short inversion-recovery time for fat suppression at b-values of 0 and 1000. The following parameters were applied: TR: 6000 ms; TE: 66–101 ms; TI: 180 ms; flip angle: 90°; FOV: 240 × 240 mm; matrix: 256 × 256; and thickness: 3 mm without gaps.

Pre-treatment ADC images were annotated by two radiologists (Dr. Zhang and Dr. Tao, both with 15 years of clinical experience), who were blinded to the patients’ treatment outcomes. The imaging annotation was completed using ITK-SNAP software version 4.1 (http://www.itksnap.org/pmwiki/pmwiki.php). Both doctors delineated all ADC imaging layers with lesions for the patients. After annotation, all extracted radiomics features were assessed with intraclass/interclass correlation coefficients (ICC), and features with ICC >0.75 were considered highly repeatable and reliable (14, 15).

### Feature extraction

Radiomics features of the patients’ ADC imaging were extracted using the open-source PyRadiomics tool (https://pyradiomics.readthedocs.io/en/latest/index.html). A total of 1288 radiomics features were extracted, including: (1) 14 shape features, (2) 252 first-order histogram features, and (3) textural features: 308 gray-level co-occurrence matrices (GLCM), 224 gray-level run length matrices (GLRLM), 224 gray-level size zone matrices (GLSZM), 70 neighboring gray-tone difference matrices (NGTDM), and 196 gray-level dependency matrices (GLDM) (16). The study design followed the Image Biomarker Standardization Initiative (IBSI) reporting guidelines (17). The GitHub address for obtaining the radiomics feature extraction configuration file (exampleMR_3mm.yaml) is https://github.com/AIM-Harvard/pyradiomics/tree/master/examples/exampleSettings.

### Radiomic feature selection and model building

To ensure the reliability and stability of data annotation, we assessed the ROI annotations by two radiology experts using the Intraclass Correlation Coefficient (ICC). The ICC value ranges from 0 to 1, where 0 indicates no consistency and 1 indicates complete consistency. A higher ICC value implies greater consistency or similarity between different observations, while a lower value indicates poorer consistency or greater differences. The ICC measures the degree of agreement between different observations or evaluators within the same group, helping to assess reliability and provide effective information in research design and data analysis. Therefore, we used ICC to select more reliable and stable features for this study.

In the ROI annotation data by two radiologists, 30 samples were randomly selected from the training set, and feature extraction was performed on the annotated data from both doctors using the PyRadiomics tool, to evaluate the reliability and stability of the annotated data. Generally, a reliability coefficient below 0.4 indicates poor reliability, while a coefficient above 0.75 indicates good reliability. Therefore, in our experiment, an ICC greater than 0.75 was chosen as the criterion for determining the reliability and stability of the extracted radiomics features. This ICC-based filtering was performed exclusively on the training set (N = 80), and the same feature set was then applied to the validation set. Ultimately, 546 radiomics features were obtained.

To further reduce feature redundancy and ensure linear correlation among features, we calculated and used the Pearson correlation coefficient between features for secondary screening. By calculating the Pearson correlation coefficient, we can evaluate the strength of linear correlation between features and thus select features that have a greater correlation with the target variable and a smaller correlation with other features. The Pearson correlation coefficient ranges from -1 to 1, where -1 indicates a perfect negative correlation, 1 indicates a perfect positive correlation, and 0 indicates no linear correlation. Generally, a correlation coefficient with an absolute value greater than 0.5 is considered to have a strong correlation. In this study, we set the screening threshold at 0.9. Features with correlation coefficients higher or lower than this threshold were screened out. For highly correlated feature pairs, one feature from each pair was randomly selected and removed to further reduce the dimensionality of the high-dimensional data. All Pearson correlation-based filtering was performed exclusively within the training set, and the retained feature set was locked before application to the validation set.

### Radiomic signature construction

To select optimal prognostic features from radiomic characteristics for PFS prediction, we employed the LASSO-Cox proportional hazards regression model with ten-fold cross-validation. Unlike logistic regression, which handles binary outcomes, the LASSO-Cox model is specifically designed for time-to-event data and applies L1-penalized regularization to the Cox partial likelihood function. This approach enables automatic selection of informative features while penalizing irrelevant ones, thereby reducing overfitting and enhancing model interpretability. The optimal penalty parameter λ was determined by minimizing the partial likelihood deviance through ten-fold cross-validation, with the 1-standard-error rule (λ.1se) selected to ensure model parsimony.

Features selected through LASSO-Cox regression were used to calculate each patient’s radiomics score as a linear combination of the selected feature values weighted by their respective Cox regression coefficients: Rad-score = Σ(feature_i × coefficient_i). This Rad-score serves as the radiomics signature, representing each patient’s relative risk of progression. Specifically, the Rad-score corresponds to the linear predictor from the Cox model, where higher scores indicate a higher hazard of PFS events.

To ensure robust feature selection and unbiased performance estimation, we implemented a nested cross-validation (NCV) framework within the training set. The outer loop (10 folds) was used for model performance evaluation, while the inner loop (10 folds) was used for LASSO-Cox parameter tuning and feature selection. This approach prevents information leakage and provides a less biased estimate of the model’s generalizability.

The best cutoff value for Rad-score, which divides patients into low-risk and high-risk groups, was determined using the ‘survMisc’ package based solely on the training set (N = 80). Ultimately, patients’ progression-free survival was stratified based on the Rad-score of each sample using this fixed training-derived threshold applied to both cohorts.

## Results

### Clinical characteristics

Using univariate Cox regression analysis, significant clinical characteristics before treatment were analyzed, with a significance level set at P<0.05. The results indicated that, aside from age and T stage (p-values = 0.0099 and 0.0386, respectively) (as shown in **Table 1**) no other clinical characteristics demonstrated significant differences.

**Table 1:** Analysis of clinical characteristics.

Variable	Coefficients	*P* value
Sex	4.740153e-11	9.915507e-01
Age	0.0007320279	0.0099389686
T	0.002029362	0.038597319
N	0.01748385	0.84480429

### Radiomic feature extraction and selection

Utilizing the PyRadiomics feature extraction tool, 1288 radiomic features were derived. Following ICC-based filtering (ICC>0.75) performed in the training set, 546 radiomic features were retained. In subsequent feature selection processes within the training set, pairs of features with an absolute Pearson correlation coefficient above 0.9 were considered to exhibit strong correlations. Following this criterion, a final set of 102 features was determined within the training set, and this feature set was locked for subsequent analysis.

Ten-fold cross-validation and the LASSO-Cox regression algorithm were applied for further feature compression, ultimately selecting six radiomic features to establish a radiomic signature score. **Table 2** lists the names and coefficients of these radiomic features. **Figure 2** show the LASSO-Cox coefficient path and cross-validation graphs, respectively. In the training cohort, the radiomic signature score model achieved a time-dependent AUC of 0.867 for 1-year PFS and 0.837 for 2-year PFS. In the validation cohort, the corresponding time-dependent AUCs were 0.737 and 0.715, respectively. The C-index of the Rad-score alone was 0.782 (95% CI: 0.710–0.854) in the training cohort and 0.743 (95% CI: 0.668–0.818) in the validation cohort.

**Table 2:** The names and coefficients of radiomic features for RAD_score.

Names	Coefficients
diagnostics_Image-interpolated_Maximum	0.009739498
diagnostics_Image-original_Minimum	-0.043349381
log-sigma-2-0-mm-3D_firstorder_90Percentile	0.04825508
wavelet-LHH_glszm_GrayLevelNonUniformityNormalized	-0.094116528
wavelet-LHH_gldm_HighGrayLevelEmphasis	0.006865134
wavelet-HLH_gldm_GrayLevelVariance	0.006078601
wavelet-HHH_glszm_ZoneVariance	-0.001663349

**Figure 2:**
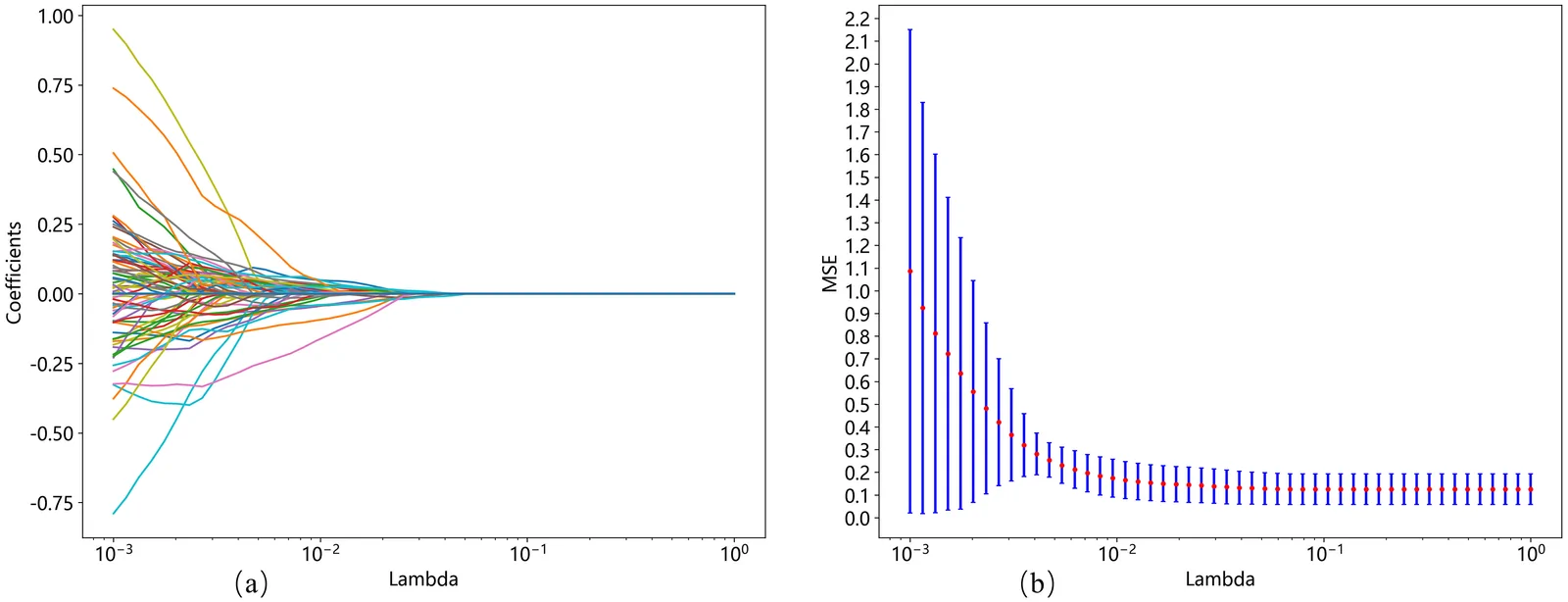
**(a)** and **(b)** show the LASSO coefficient path and cross-validation graphs, respectively.

### Prediction nomogram development and validation

Using the radiomics signature score and independent clinical predictors identified by univariate Cox regression (age and T stage, p< 0.05), a radiomics nomogram model was constructed (as shown in **Figure 3**). The nomogram was built using a multivariable Cox proportional hazards model, with the Rad-score and clinical predictors as covariates. The nomogram provides estimated probabilities of PFS at 1 and 2 years post-treatment.

**Figure 3:**
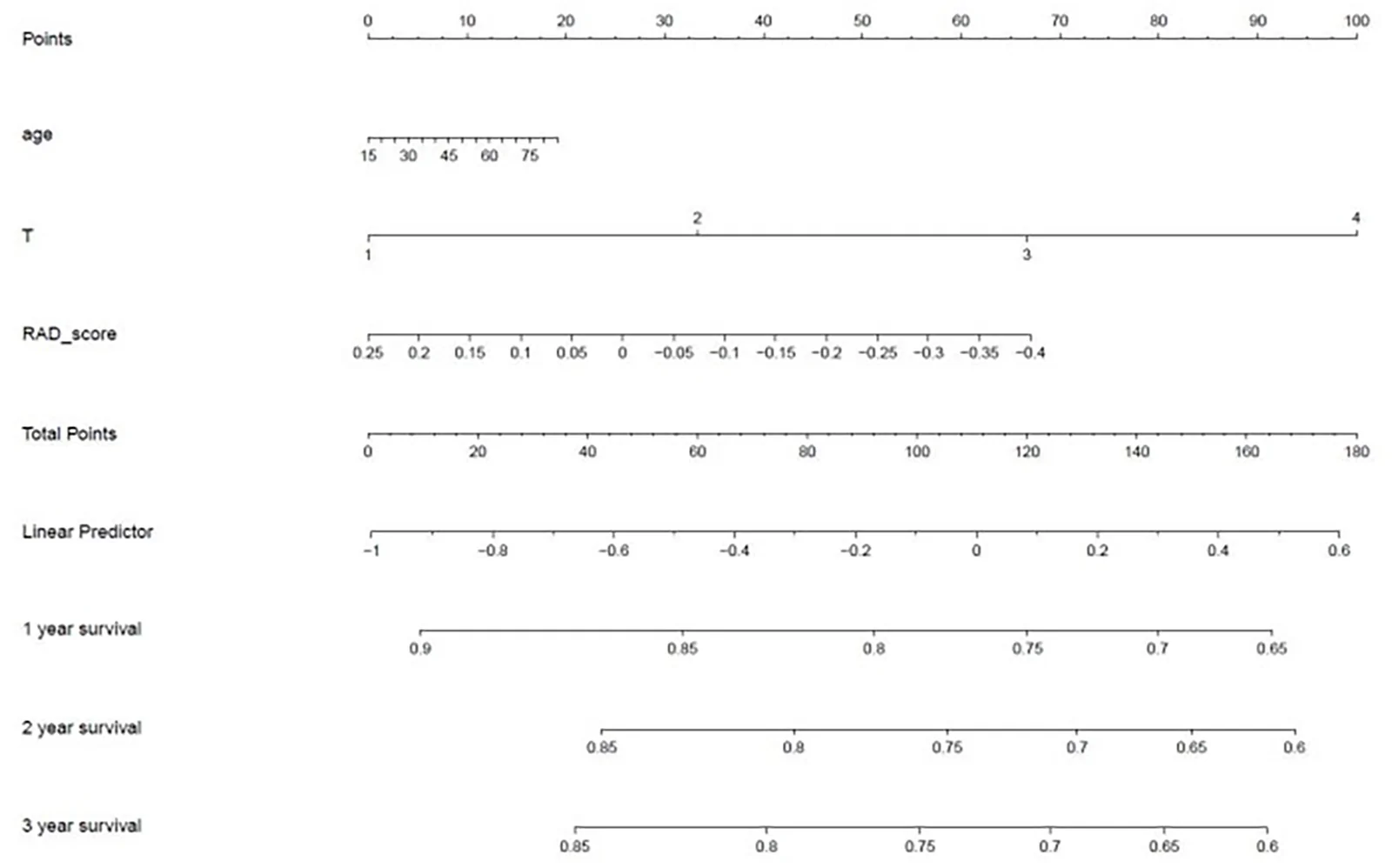
Randomics nomogram incorporating the rad_score with all independent clinical predictors.

In both the training and test sets, there were significant differences in the radiomic Rad-score between patients with and without progression events, with log-rank p-values of 0.006 and 0.023, respectively (as shown in **Figure 4**). The C-index of the nomogram model, constructed from clinical features and the Rad-score, was 0.904 (95% CI 0.816-0.992) in the training set and 0.858 (95% CI 0.690-1.000) in the test set, which was higher than those of the models based on clinical features alone or radiomic score alone. The time-dependent ROC curves for the three models in the training and test datasets at 1-year and 2-year prediction horizons are displayed in **Figure 5**.

**Figure 4:**
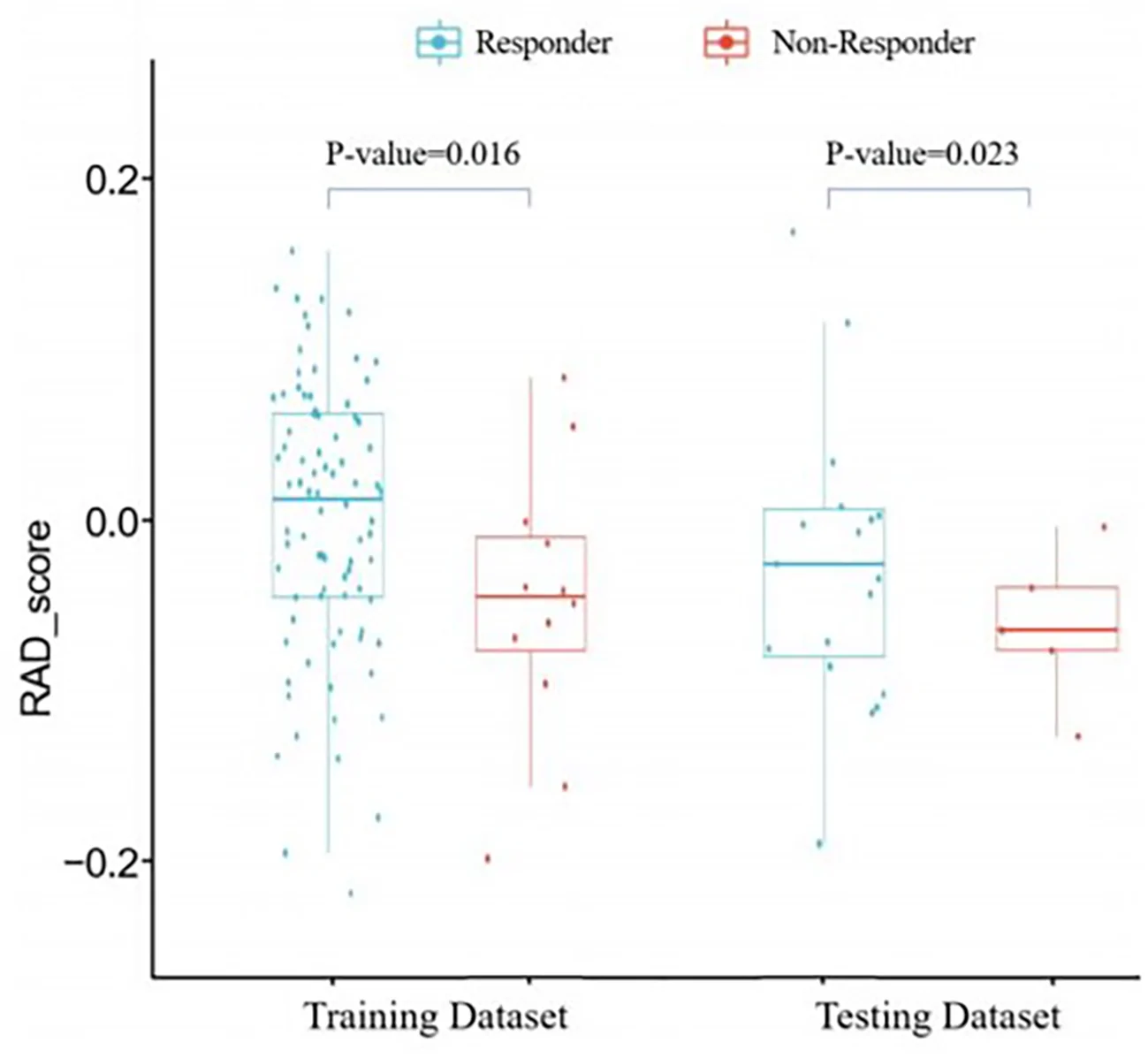
Box plot of RAD_score for sensitive and non-sensitive patients in the training and validation cohorts.

**Figure 5:**
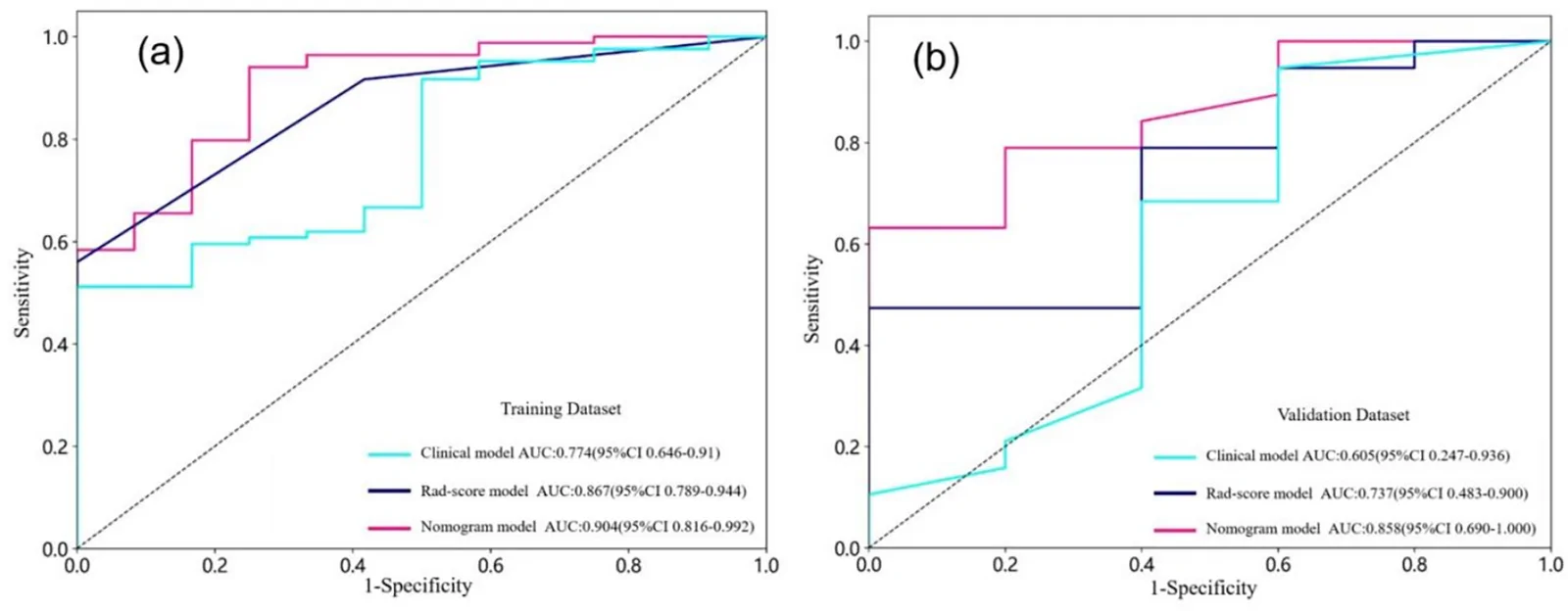
**(a)** ROC curves comparison for the radscore, clinical model, and radiomics nomogram in training cohorts **(b)** ROC curves comparison for the radscore, clinical model, and radiomics nomogram in validation cohorts.

As shown in **Figure 6**, the calibration curves of the radiomics nomogram model in both the training and test sets indicate good agreement between the predicted 1-year PFS probabilities and actual observed PFS rates in patients with HPSCC, with p-values of 0.537 and 0.389 from the Hosmer-Lemeshow test, respectively (not significant, indicating good calibration). The decision curve analysis of the nomogram model (shown in **Figure 7**) demonstrates that the radiomics nomogram model exhibits superior diagnostic performance in both the training and validation cohorts compared to the clinical model and the radiomic score model.

**Figure 6:**
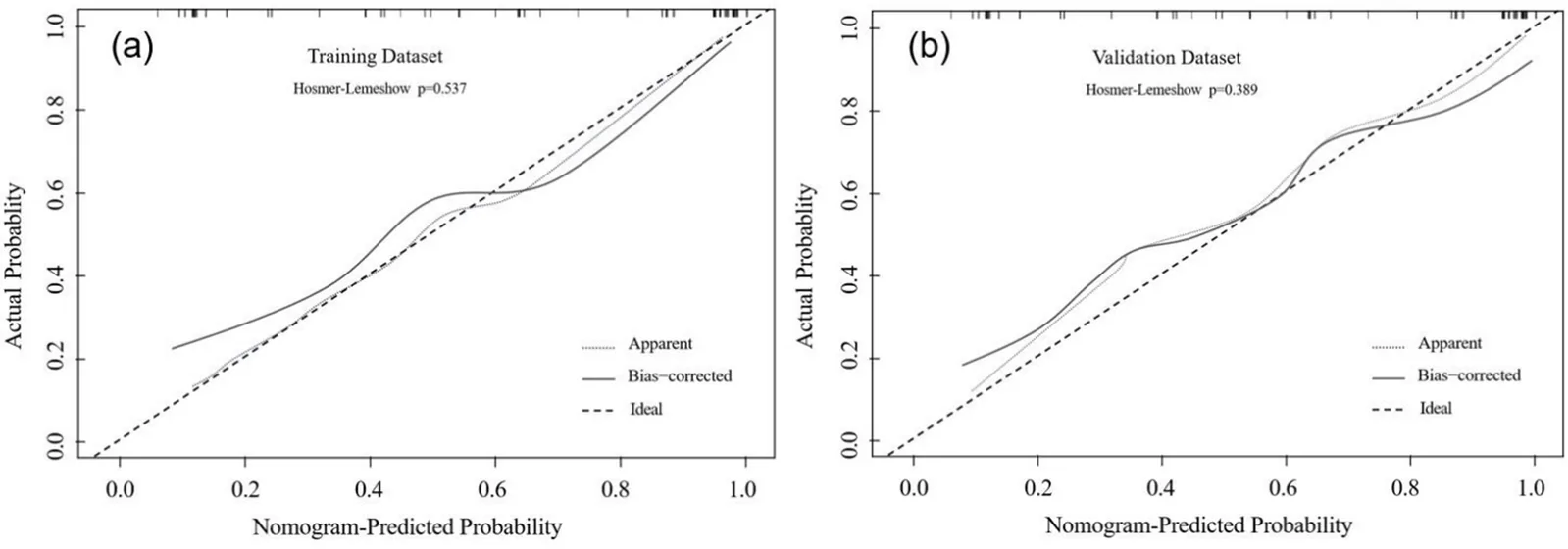
**(a)** Calibration curves for the radiomics nomogram in training cohort. **(b)** Calibration curves for the radiomics nomogram in validation cohorts. The solid line represents actual performance of the radiomics nomogram. The dashed line represents a perfect prediction by an ideal model. A closer fit to the dashed line demonstrates a better prediction.

**Figure 7:**
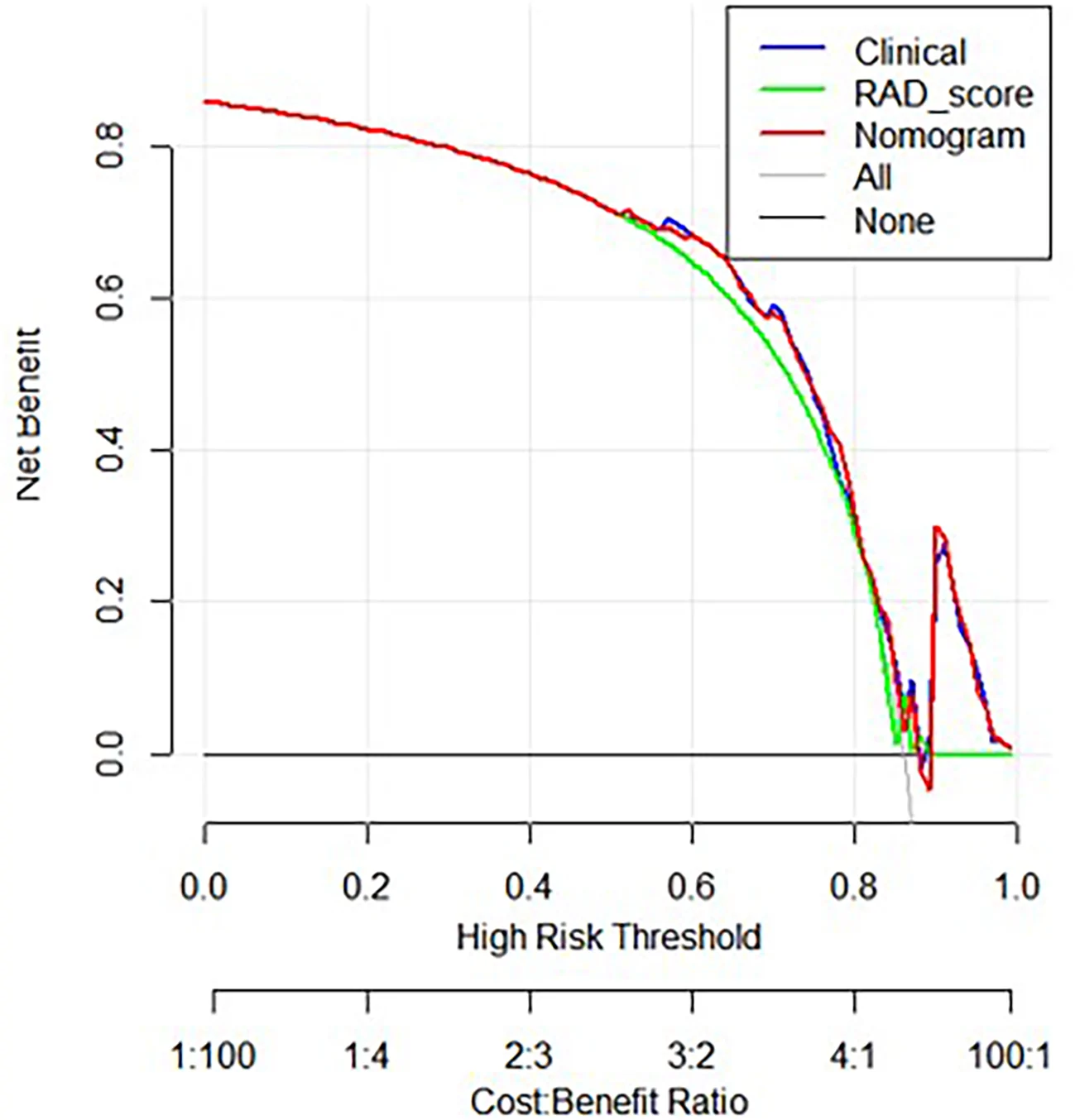
Decision curve analysis for the radiomics nomogram.

### The value of radiomics signature in the stratification of risk of progression-free survival

The optimal cutoff value for Rad-score, which divides patients into low-risk and high-risk groups, was determined to be 0.025 by the ‘survMisc’ package using the training set only. This fixed cutoff value was then applied to the validation set for risk stratification. **Figure 8** shows the Kaplan-Meier (KM) survival curves for the low- and high-risk PFS groups in the training set (p=0.006) and validation set (p=0.023), with median survival times of 20.5 months and 9.7 months, respectively in the combined cohorts. The log-rank test p-value between the two groups was 0.006 in the training set, indicating that the Rad-score has good stratification ability for patients’ progression-free survival and can provide a reference for clinical assessment.

**Figure 8:**
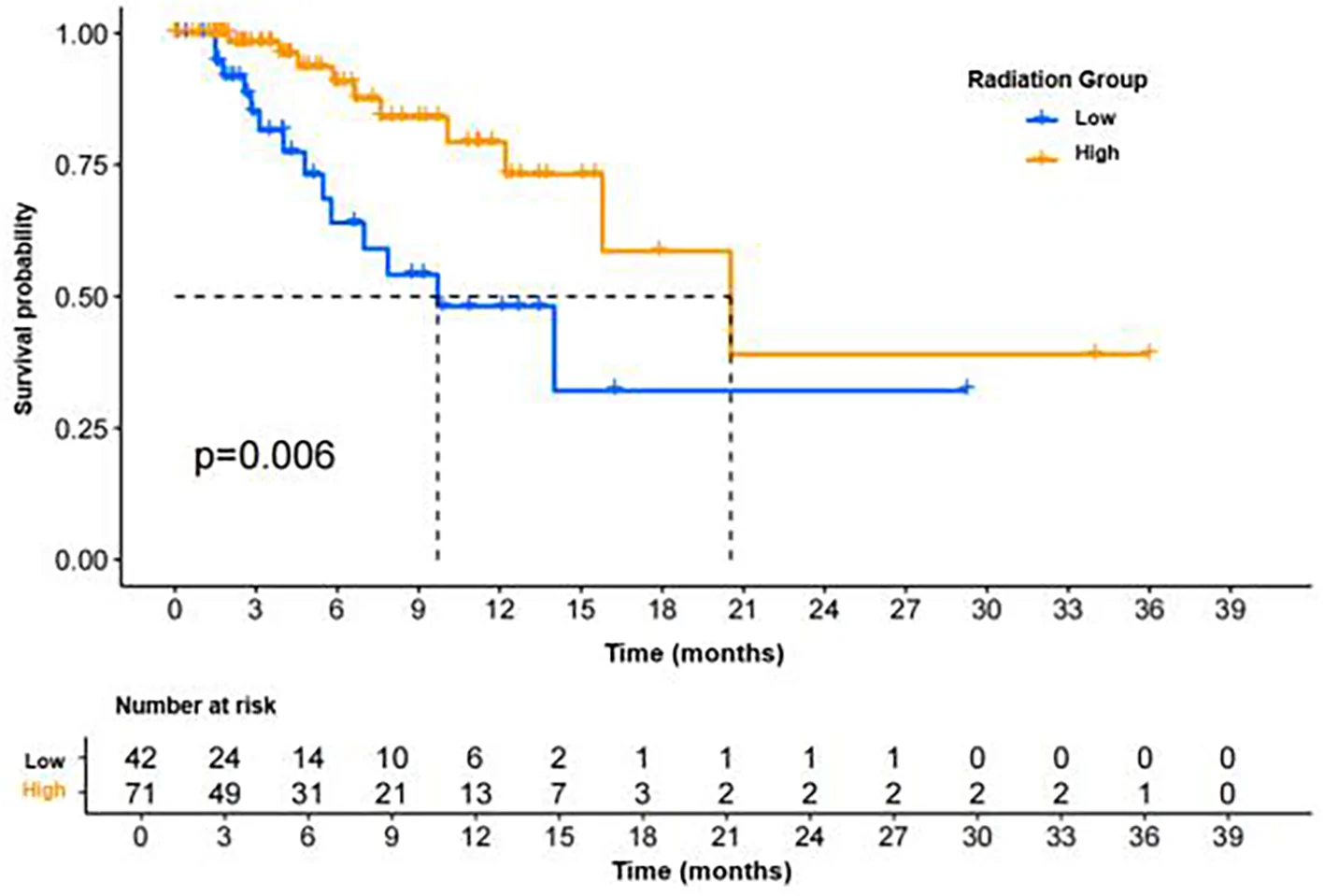
PFS analysis stratified by the optimal cut off value of radiomics signature.

## Discussion

In this study, our results demonstrated that Rad-score based on the LASSO-Cox showed the highest ability to predict PFS. Furthermore, we constructed a radiomics-clinical nomogram that combined clinical T-staging and Rad-score, and evaluated it in both training and validation cohorts. This combined model showed better predictive capability for PFS than either the radiomics nomogram or the clinical nomogram alone.

HPSCC is a malignancy tumor with poor clinical prognosis in head and neck cancers (HNC) (18). While the prognostic potential of CT-based radiomic features in head and neck cancer has been confirmed (19, 20), the predictive performance of radiomic models based on MRI-ADC maps for specific types, particularly HPSCC, requires further investigation. Liu et al (15)investigated the prognostic value of CT-based radiomic features in predicting the risk of progression in HPSCC with induction chemotherapy. Chen et al (21) evaluated 5-year overall survival (OS) in patients with advanced HPSCC (stages III–IV) using T1-MRI based radiomics, achieving a C-index of 0.78. It is important to note that MRI artifacts can sometimes influence radiomic features extraction. Conventional studies commonly employ multi-sequence imaging for comprehensive tumor characterization, but this may incur morphological noise unrelated to essential biological behavior. Conversely, ADC sequences enable direct quantification of water diffusivity, and their radiomic metrics are directly linked to tumor microenvironmental parameters such as cell density and hypoxia (22). To ensure uniform image quality, we only used images with no visible artifacts in the tumor area. Among MRI sequences, ADC maps are commonly used for visual guidance in tumor delineation (23), and radiomic features derived from ADC maps have been proved to have strong predictive power (24, 25). A latest study enrolling 120 patients with hypopharyngeal carcinoma revealed that a radiomic model derived exclusively from ADC images could predict chemoradiotherapy response with an AUC of 0.947 on the validation cohort. More strikingly, the incorporation of deep learning algorithms boosted the predictive performance to an unprecedented AUC of 1.000 (26). Therefore, we selected pretreatment ADC maps to construct our radiomics model and predict patients’ PFS, achieving predictive performance similar to previously reported results (14, 25).

There have been studies demonstrated the effectiveness of ADC in predicting local failure of HNC. For instance, Lombardi et al. showed that the value of ADC before CRT could predict disease recurrence (27). In our study, 1288 radiomic features were extracted from each patient, and six most significant features were identified. Calculating the AUC to assess the sensitivity and specificity of a predictive model based on these six features showed that ADC-based radiomic features could accurately identify patients at risk for recurrence. The results of our survival analysis confirmed that the model estimated a poorer prognosis for patients at high risk of recurrence than for those at low risk, underscoring the clinical value of our predictive model. Meanwhile, previous studies have demonstrated the predictive performance of radiomic features based on ADC maps in prostate cancer (28), cervical cancer (29), and rectal cancer (30).

Our radiomic model comprised six key features selected from high-dimensional 3D texture features of the tumor, aimed at providing prognostic information for risk stratification of tumor progression. First-order features describe the distribution pattern of gray-level pixel values across the tumor volume. In particular, the maximum and minimum ADC values reflect the overall density extremes of the lesion, which are linked to hemodynamic parameters and have been shown to predict chemotherapeutic efficacy (30). Our study confirmed that both the maximum and median ADC values were predictive of PFS in HPSCC patients undergoing CCRT. Regarding textural features, previous radiomic studies on hypopharyngeal cancer have reported that a higher gray-level non-uniformity value indicates greater tumor heterogeneity (14, 31), and this association with worse prognosis has been further corroborated by multiple investigations (32, 33). This feature quantitatively assesses heterogeneity by evaluating the uniformity of gray-level zone size distribution across the tumor; elevated values denote more diverse and irregular zone patterns, reflecting pronounced intra-tumoral variations in cellular density, necrotic architecture, and microenvironmental composition. In line with these findings, our research demonstrated that the normalized gray-level non-uniformity derived from ADC maps was capable of predicting PFS in this patient cohort.

A radiomics study based on MR reported that first-order and second-order features were associated with potentially indicating a better prognosis (29). The radiomic features extracted in our study included the above features, which were conformity with previously published studies. Additionally, our findings revealed that Glszm_ZoneVariance was a radiomic feature related to PFS, not discovered in previous studies. Glszm_ZoneVariance measures the dispersion of zone sizes within the matrix. Higher values indicate marked size disparities among textural regions, reflecting internal architectural inhomogeneity-an important predictor of radiation efficacy (34). Higher spatial structural heterogeneity is conventionally linked to greater tumor aggressiveness and therapeutic resistance. The differences between our results and previous studies can be attributed to the differences in study design. Unlike previous studies based on T1 and T2 MRI images, our study focused on ADC images to obtain imaging features. However, the biological explanation of radiomic features need further investigation.

When the nomograms designed in our study were verified, there was a good consistent between the predicted results and the observed 1-, 2-, and 3-year PFS. Multiple studies have confirmed that old age, large tumors and advanced stage of lymph nodes are adverse factors affecting the prognosis of HPSCC patients (35–37). The nomogram in our study combines T-stage, age, and important radiomic features to predict the risk of hypopharyngeal squamous cell carcinoma progression, while developing individualized treatment strategies for patients in advance. Based on the risk stratification, we have delineated hypothetical clinical decision-making algorithms. For the High-Risk group, we discuss the possibility of intensifying treatment by adding immunotherapy (ICIs) or induction chemotherapy by MDT (Multi-Disciplinary Team) members. For the Low-Risk group, we suggest maintaining standard chemoradiation with potentially extended imaging intervals to avoid overtreatment. Therefore, our radiomics-clinical model provides an important biomarker to predict the risk of progression and to pre-screen patients at risk for early progression in HPSCC patients receiving radical CRT.

This study has several limitations. First, patients in our study were from a single center, selection bias existed. Second, we focused solely on features extracted from ADC maps, excluding other MRI sequences. Third, the sample sizes are relatively small, necessitating a larger prospective cohort to validate our findings.

## Data Availability

The original contributions presented in the study are included in the article/supplementary material. Further inquiries can be directed to the corresponding authors.
